# *Helicobacter pylori* antibiotic resistance profile in Chinese children with upper gastrointestinal symptoms and a literature review for developing personalized eradicating strategies

**DOI:** 10.3389/fphar.2024.1392787

**Published:** 2024-06-03

**Authors:** Danli Zhou, Wuyu Wang, Lan Gu, Meiling Han, Wujuan Hao, Junfeng Huang, Qiong Lin, Yan Wang

**Affiliations:** ^1^ Department of Pharmacy, Affiliated Children’s Hospital of Jiangnan University, Jiangsu University, Wuxi, China; ^2^ Department of Burns and Plastic Surgery, Affiliated Children’s Hospital of Jiangnan University, Jiangsu University, Wuxi, China; ^2^ Department of Gastroenterology, Affiliated Children’s Hospital of Jiangnan University, Jiangsu University, Wuxi, China

**Keywords:** children, *Helicobacter pylori*, eradication, resistance, susceptibility testing

## Abstract

**Background:** *H. pylori* (*Helicobacter pylori*) infections typically occur in early childhood. Although the prevalence of *H. pylori* in children is lower than that in adults, the eradication rate of this infection in children is relatively low because of resistance. In this study, we analyzed personalized treatment strategies to achieve treatment goals based on *H. pylori* resistance characteristics. This retrospective single-center study was conducted between January 2019 and December 2022 and enrolled 1,587 children who presented with upper gastrointestinal symptoms and underwent endoscopy. *H. pylori* culturing and antimicrobial susceptibility testing were performed.

**Results:** Culture-positive results for *H. pylori* were obtained in 535 children. The resistance rates to clarithromycin (CLA), metronidazole (MET), and levofloxacin (LEV) were 39.8%, 78.1%, and 20.2%, respectively. None of the isolates were resistant to tetracycline (TET), amoxicillin (AMO), or furazolidone (FZD). Double resistance rates to CLA + MET, CLA + LEV, and MET + LEV were 19.1%, 3.0%, and 5.8%, respectively. Notably, triple-resistant to CLA + MET + LEV was 9.7%. Based on susceptibility tests, individualized triple therapy [proton pump inhibitor (PPI) +AMO + CLA/MET] was selected for 380 children with *H. pylori* sensitive to MET and/or CLA. In 155 children resistant to CLA and MET, bismuth-based quadruple therapy was recommended; for unable to receive bismuth, concomitant therapy was recommended for 14 children (<8 years of age); triple therapy with TET was recommended for 141 children (>8 years of age), with 43 children (>14 years of age) requiring FZD rather than TET.

**Conclusion:** Resistance to *H. pylori* in Chinese children was relatively poor. Personalized therapy regimens should be based on susceptibility tests and avoided factors associated with treatment failure.

## 1 Introduction

*Helicobacter pylori* (*Helicobacter pylori*), identified by Warren and Marshall over 40 years ago, remains a common chronic infectious disease worldwide (Seo et al., 2020). The overall global prevalence of *H. pylori* is predicted to exceed 50% (Katelaris et al., 2023). With recent socioeconomic development and environmental advances, the prevalence of *H. pylori* has markedly declined (Peleteiro et al., 2014). Its prevalence in China decreased from 58.3% between 1983 and 1994 to 40% between 2015 and 2019 (Ren et al., 2022). Similar trends were observed in Japan, with an adult infection rate of 72.7% in 1974, which decreased to 40% by 2014 (Ueda et al., 2014). In Nepal, The prevalence of *H. pylori* infection in children has also decreased from 39% before 2000 to 26% in 2010 (Mehata et al., 2021). Moreover, *H. pylori* infection rates vary among children of different ages and increase with age (Borka et al., 2022), although this trend is not observed in adults (Ren et al., 2022).

Most *H. pylori* infections are acquired during early childhood (Okuda et al., 2019). Long-term infection may lead to serious diseases, such as cancer and peptic ulcers. Consensus guidelines state that adults with *H. pylori* infection should initiate eradication therapy (Romano et al., 2022; Garcés-Duran et al., 2023). In contrast, unlike adults, children with *H. pylori* infection rarely develop severe complications, and early childhood infection may lead to later immune benefits (Melby et al., 2020; Martin-Nuñez et al., 2021). Therefore, “test to treat” strategies are not applicable to children (Malfertheiner et al., 2017; Ding et al., 2022), and the decision to initiate *H. pylori* eradication treatment in children requires caution.

An eradication rate of 90% is required for *H. pylori* to prevent antibiotic resistance and reduce risks associated with rescue treatment (Graham and Fischbach, 2010; Alfaro et al., 2023). In the 1990s, triple therapy with a proton pump inhibitor (PPI) + amoxicillin (AMO) + clarithromycin (CLA) was used to achieve an eradication rate of 98%; however, this rate recently decreased to 70%, particularly in children (Malfertheiner et al., 2007; Agudo et al., 2010). Various factors lead to eradication failure, such as a high bacterial load, inappropriate treatment or antibiotic concentrations, host mucosal immunity, and extragastric sources of bacteria (Moghadam et al., 2021). Unlike in adults, personalized treatment is crucial for children because age groups limit the flexibility of antibiotic choices. In 2016, the European and North American Societies of Pediatric Gastroenterology, Hepatology, and Nutrition (ESPGHAN, NASPGHAN) recommended that tailored therapy for naïve patients should be based on antimicrobial susceptibility (Jones et al., 2017). Even using these measures, recent susceptibility-guided therapy studies reported that the eradication rate has not reached 90% (Luo et al., 2020; Van Thieu et al., 2021). Hence, apart from individualized antibiotics, the treatment regimen for *H. pylori* must be improved, and personalized decisions should be made regarding other drugs and treatment measures.

In this study, we designed individualized treatment strategies to achieve treatment goals based on the resistance characteristics of *H. pylori* in children from different regions. The tailored design not only includes the selection of antibacterial drugs based on antimicrobial susceptibility tests, but also includes PPI optimization based on the characteristics of the Chinese population, compliance improvement, and probiotic combination. This study provides a foundation for further clinical research and treatment decisions.

## 2 Materials and methods

### 2.1 Patients and ethical considerations

In this retrospective study, we evaluated 1,587 children who presented with upper gastrointestinal symptoms and underwent gastrointestinal endoscopy at the Gastroenterology Clinic of the Affiliated Children’s Hospital of Jiangnan University between January 2019 and December 2022. Exclusion criteria were as follows: patients under 1 year old; those who used antibiotics, bismuth agents, or acid-inhibitory drugs in the previous month; and those with other serious diseases. For children who underwent endoscopy more than once, only the first positive test result was included. This study was approved by the Ethics Committee of the Affiliated Children’s Hospital of Jiangnan University (Approval Number: WXCH 2023-04-070). Informed consent was obtained from the children’s guardians before gastroscopy.

### 2.2 *Helicobacter pylori* culture

Gastric mucosa samples were collected from within 5 cm of the curvature of the pylorus antrum, transferred into sterile vials containing brain heart infusion broth (Oxoid, Basingstoke, UK) supplemented with 20% glycerol, and stored at 4 °C. The samples were transferred to Zhiyuan Medical Inspection Institute for *H. pylori* culture and antibiotic susceptibility testing. *H. pylori* test results from this company have also been reported in other Chinese studies (Shu et al., 2022). The homogenates of the gastric mucosa samples were inoculated on a plate containing 5% fresh fibrous defibrinated sheep blood and maintained at 37 °C under micro-aerobic conditions (5% O_2_, 10% CO_2_, and 85% N_2_). Colony growth was observed after 48–72 h. Suspected bacterial colonies were stained for microscopic examination. Gram-negative bacteria were observed under a microscope, those with a curved or gull-shaped morphology and positive activity testing (including oxidase, catalase, and urease) were determined to be *H. pylori*-positive. If no suspicious colonies were found, the cultivation time was extended to 7 days. If any strain was found, it was determined to be *H. pylori*-positive through microscopic examination.

### 2.3 Antimicrobial susceptibility testing

We performed sensitivity testing of *H. pylori* to six antibiotics [CLA, AMO, tetracycline (TET), furazolidone (FZD), metronidazole (MET), and levofloxacin (LEV)] using the agar dilution method. According to a 2.0 McFarland standard, each suspension (2 µL) was inoculated onto an antibiotic plate containing 5% defibrillated sheep blood and maintained at 37 °C in a tri-gas incubator in a microaerobic environment for 3 days. Drug sensitivity was evaluated based on the growth status of the colonies at the inoculation site. Resistance to CLA, AMO, FZD, MET, LEV, and TET was defined as minimal inhibitory concentrations of >1, >2, >2, >8, >2, and >2 μg/mL, respectively, according to the Clinical and Laboratory Standards Institute document M100-S18 and other previously published data (Peretz et al., 2014; Shu et al., 2022). The standard strain of *H. pylori* ATCC43504 (NCTC11637) was used as a control, and each experiment was repeated twice in parallel. We retrospectively analyzed the test results of these children at that time.

### 2.4 Statistics

We performed statistical analysis using the SPSS statistical software package version 26.0 (SPSS, Inc., Chicago, IL, USA). The outcome variable was the frequency count and expressed as the rate (%) of resistance to the antibiotics. Chi-squared (χ^2^) test was used to investigate associations between sex, age, and antibiotic resistance among pediatric patients. Statistical significance was set at *p* < 0.05.

## 3 Results

### 3.1 Epidemiology and resistance of *Helicobacter pylori* in Chinese children

Gastric biopsies were collected from 1,587 children and adolescents for *H. pylori* culture. In total, 535 (33.7%) patients with *H. pylori* were enrolled, including 294 (55.0%) boys and 241 (45.0%) girls, with no difference in *H. pylori* prevalence between sexes (Table 1). The infection rate of *H. pylori* in Chinese children varied slightly among the three age groups, in descending order: 13–18 years, 7–12 years, and 1–6 years (*p* < 0.05) (Table 1).

**TABLE 1:** Demographic characteristics of the patients and *Helicobacter pylori* culture of 1,587 samples obtained from patients from 2019 to 2022.

Variables	*H. pylori* positive	*H. pylori* negative	*H. pylori* positive rate n (%)	*p*-value
Sex				
Male	294	571	865 (34.0)	0.8 (0.065)
Female	241	481	722 (33.4)	
Age groups, year				
1–6	28	174	202 (13.9)	—
7–12	273	591	864 (31.6)	0.001^a^
13–18	234	291	525 (44.6)	0.001^b^, 0.001^c^

^a^ Comparison of the 7–12 and 1–6-year-old groups.

^b^ Comparison of the 13–18 and 7–12-year-old groups.

^c^ Comparison of the 13–18 and 1–6-year-old groups.

Of the 535 *H. pylori*-positive patients, 9.5% (51/535) were not resistant to any of antibiotics. The total resistance rates of *H. pylori* to CLA, MET, and LEV were 39.8% (213/535), 78.1% (418/535), and 20.2% (108/535), respectively (Table 2). None of the strains were resistant to AMO, FZD, or TET. Additionally, 52.7% (282/535) of the strains were resistant to CLA, MET, or LEV, and 37.8% (202/535) were resistant to more than one antibiotic; 27.9% (149/535) were double-resistant, and 9.9% (53/535) were triple-resistant. Notably, double resistance rates to CLA + MET, CLA + LEV, and MET + LEV were 19.1%, 3.0%, and 5.8%, respectively (Table 2). Moreover, only 60 children infected with *H. pylori* were sensitive to MET and CLA. Sex was not associated with antibiotic resistance. Considering the low infection rate of *H. pylori* in children under the age of 6 years, the one to six and 7–12 years groups were merged into a 1–12 years group for age stratification to analyze resistance to various antibiotics. Our results indicate that age is associated with resistance to MET and LEV, but not to CLA (Table 3). The MET resistance rate increased with age, whereas LEV resistance rate decreased.

**TABLE 2:** Results of antibiotics susceptibility tests of 535 *Helicobacter pylori* strains isolated from 2019 to 2022.

Susceptibility test results	Resistant strains	
	N	%
Total resistance		
CLA	213	39.8
MET	418	78.1
LEV	108	20.2
Single resistance		
CLA	42	7.9
MET	232	43.4
LEV	8	1.5
Double resistance		
CLA + MET	102	19.1
CLA + LEV	16	3.0
MET + LEV	31	5.8
Triple resistance		
CLA + MET + LEV	53	9.9

CLA, clarithromycin; MET, metronidazole; LEV, levofloxacin.

**TABLE 3:** Factors associated with *Helicobacter pylori* resistance to CLA, MTZ, and LEV in pediatric patients.

Variables	Total	CLA	*p*-value	MET	*p*-value	LEV	*p*-value
		R (%)		R (%)		R (%)	
Sex							
Male	294	110 (37.4)	0.2	231 (78.6)	0.78 (0.074)	55 (18.7)	0.4
Female	241	103 (42.7)		187 (77.6)		53 (22.0)	
Age groups, year							
1–12	301	128 (42.5)	0.2	223 (74.1)	0.01	74 (24.6)	0.004
13–18	234	85 (36.3)		195 (83.3)		34 (14.5)	

CLA, clarithromycin; MET, metronidazole; LEV, levofloxacin; R, resistance; S, susceptibility.

## 4 Discussion

### 4.1 Antibiotic resistance in *Helicobacter pylori*

In this study, we analyzed the current resistance of *H. pylori* in children in the local area. Our study showed that approximately 33.7% of children are infected with *H. pylori*, which is not substantially different from the global infection rate in children (Seo et al., 2020). We cited an overall resistance rate to CLA (39.8%), MET (78.1%) and LEV (20.2%) with 535 *H. pylori* pediatric strains isolated in Southeast China. In our study, the resistance rate of LEV is 20.2%, which is lower than that of CLA and MET. However, as quinolone antibiotic, LEV can cause arthropathy and bone/cartilage disease in some species of animals. Its safety for children has not been established, and it is forbidden to use under 18 years old in China. In our research, 19.1% of children with *H. pylori* infection were resistant to CLA and MET.

Antimicrobial resistance is the most common cause of treatment failure in *H. pylori* infection. The MET resistance rate of *H. pylori* ranges from 10.1% to 91% in pediatric patients (Borka et al., 2023). Children in East and Southeast Asia and Africa exhibit high resistance to MET (Borka et al., 2023). We demonstrated that MET resistance among children was 78.1%, which was much higher than the regional MET resistance rate reported in Jiangxi province (42%) (Liu et al., 2019) and Taiwan (26.8%) (Lu et al., 2019); however, these results were comparable to the reported rates of 75.2% in Zhejiang province (Li et al., 2017) and 71.3% in southeast China (Hu et al., 2018). Moreover, resistance to MET in this study markedly increased with age. Benefiting from its restricted use, the MET resistance rate was not high in Japan. In triple therapy, the eradication rate in children using MET was over 95% (Malfertheiner et al., 2017; Ding et al., 2022). However, the results of our study and those of other studies on Chinese children showed high MET resistance, although MET use in standard triple therapy is not applicable in Chinese children (Li et al., 2017; Hu et al., 2018).

As a macrolide antibiotic, CLA is stable and available in the low-pH environment of the stomach. Recently, the CLA resistance rate has steadily increased in some regions and countries (Abadi, 2017), which may be related to the widespread use of macrocyclic antibiotics in children with respiratory infections. Eradication rates in areas with high CLA resistance are lower than those in areas with low CLA resistance (Liang et al., 2012). In a study performed in Wenzhou, Zhejiang province, China, when CLA resistance reached 26.12%, the efficacies of triple therapy and bismuth-containing quadruple therapy based on the antibiotic susceptibility test were 67.32% and 68.49%, respectively (Pan et al., 2020). Studies in Vietnam revealed increased resistance to CLA, with rates of 50.9% in 2006 (Le et al., 2022), 84.6% in 2012 (Le et al., 2022), 92.1% in 2021 (Van Thieu et al., 2021), and 81% in 2022 (Borka et al., 2023). The success rate of treatment based on antimicrobial testing is 75% in Vietnam (Nguyen et al., 2012). Nevertheless, in our study, CLA resistance among 535 *H. pylori* strains was as high as 39.8%, which was much higher than the reported rate of 16.4% from 2009 to 2015 in Zhejiang, of our neighbored city (Li et al., 2017). A similar trend of CLA resistance has also emerged in Europe, where they have implemented the intervention program of macrolide drugs, particularly for respiratory tract infections in children (Megraud et al., 2021). Therefore, we need to pay attention to the rational use of macrolide antibiotics in children to reduce drug resistance.

The resistance of *H. pylori* in Chinese children is critical. Therefore, individualized treatment based on a susceptibility test may be challenging for the doctor.

### 4.2 Analysis of *Helicobacter pylori* in Chinese children based on antimicrobial susceptibility testing

#### 4.2.1 Sensitivity to CLA and/or MET

Age restrictions exist for certain antibacterial agents, therefore, antibacterial agents for *H. pylori* eradication in children include AMO, CLA, MET, FZD, and TET. Currently, standard triple, sequential, concomitant, and bismuth-based quadruple therapies are recommended to eradicate *H. pylori*. A study of Chinese children showed that the eradication rates of standard triple, sequential, concomitant, and bismuth-based quadruple therapy were 74.1%, 69.5%, 84.6%, and 89.8%, respectively (Zhou et al., 2020). In the previous study, bismuth-based therapy, but not sequential or concomitant therapy, was superior to triple therapy (Zhou et al., 2020). However, the authors did not consider antimicrobial susceptibility or adherence. Children susceptible to CLA and MET received either 10-day sequential or triple therapy, whereas those resistant to CLA or MET received 10-day triple therapy (Kotilea et al., 2017). The eradication rates of sequential treatment, triple therapy with CLA, and triple therapy with MET were 92%, 77%, and 70%, respectively (Kotilea et al., 2017). Furthermore, eradication was achieved in 86.8% and 92.3% of children who received at least 90% of the prescription drugs in triple therapy containing CLA or MET, respectively (Kotilea et al., 2017). Similar findings were reported in children from Japan, emphasizing that triple therapy, with good adherence to the prescribed drugs, can achieve a primary eradication rate of 97.7%, according to the antimicrobial susceptibility test (Miyata et al., 2021). Currently, most countries consider personalized triple therapy as the preferred treatment for children infected with sensitive *H. pylori* (Jones et al., 2017; Kato et al., 2020; Subspecialty Group of Gastroenterology et al., 2023). ESPGHAN/NASPGHAN also recommends sequential therapy for children sensitive to both MET and CLA (Jones et al., 2017). According to Schwarzer et al. (2016), the eradication of sequential therapy in no resistance and single resistance were 85.8% and 72.6%, respectively, based on the use of esomeprazole and high compliance. However, sequential therapy for pediatric patients involves exposure to three antibacterial drugs, and the medication regimen for sequential therapy is complex, which can affect children’s treatment compliance. Therefore, most current guidelines do not recommend sequential therapy as first- or second-line therapy, particularly for adults (Chey et al., 2017; Malfertheiner et al., 2017; Kato et al., 2019; Kato et al., 2020; Romano et al., 2022). Notably, children with single resistance have a higher risk of treatment failure than those susceptible to CLA and MET (Le et al., 2023). The eradication with single resistance is usually below 90% (Schwarzer et al., 2016; Le et al., 2023). This may be because the resistant site is omitted during sampling, and thus does not represent the actual resistance.

Based on these findings and considering the resistance to CLA and MET in Chinese children, PPI + AMO + CLA should be selected for *H. pylori*-infected children who are sensitive to MET and CLA, and medication guidance should be provided to improve compliance and to ensure that more than 90% of prescriptions are followed. For who are sensitive to MET or CLA, the eradication rate is reduced, and it is possible to consider initiating bismuth-based quadruple therapy recommended by ESPGHAN/NASPGHAN (Jones et al., 2017).

#### 4.2.2 Resistance to CLA and MET

According to the ESPGHAN/NASPGHAN guidelines, triple therapy with PPI-AMO at high doses for 14 days or concomitant therapy, or bismuth-based quadruple therapy is recommended for patients who are resistant to CLA and MET. However, research on the use of high-dose AMO in children is limited. The eradication rate achieved using a combination of esomeprazole, high-dose AMO, and MET in children with dual resistance did not exceed 75% (Schwarzer et al., 2011), even when using esomeprazole, which is not strongly influenced by CYP2C19 (Schwarzer et al., 2011). A similar conclusion was found in another study conducted in Vietnam; for children showing resistance to MET and CLA, the eradication rate of using high-dose AMO therapy was only 32.6%, which was lower than that obtained using the standard dose of AMO (60.6%, *p* = 0.015) (Van Thieu et al., 2021; Ding et al., 2022). In these studies, high-dose AMO had a poor effect on children with multi-antibiotic-resistant *H. pylori.*

Combination and sequential therapies have the same drawbacks, exposing the three antimicrobial drugs and affecting treatment compliance. Combination therapy does not have a superior eradication rate and, therefore, is not recommended when other treatment regimens are available. Bismuth compounds may play two main roles: heavy metals possess antimicrobial properties and interfere with *H. pylori* attachment when these electron-dense bodies are deposited on the cell surface (Pacifico et al., 2012), and bismuth compounds have a synergistic effect with antibiotics in overcoming multi-antibiotic resistance (Ko et al., 2019).

In China, TET and FZD are recommended for treating *H. pylori* infections in children. We detected no strains resistant to TET or FZD. The resistance rate of Chinese children to these two antibiotics is low. Therefore, TET and FZD can be chosen as substitutes for CLA and MET, respectively, to overcome dual resistance. However, because of the genotoxic and carcinogenic effects of FZD and tooth calcification caused by TET, these drugs are prescribed for children older than eight and 14 years, respectively. However, there are age limitations for the administration of TET and FZD, which have insufficient efficacy in treating *H. pylori* infection in children.

ESPGHAN/NASPGHAN guidelines recommend alternative drugs for children with AMO allergies. Limiting antibiotic use leads to lower antibiotic resistance development. Therefore, the selection of new antibiotics to overcome antibiotic resistance may lead to antibiotic resistance. Most clinical physicians and patients evade bismuth-containing therapy because of adverse reactions. The bismuth compounds currently used are insoluble inorganic salts with a systemic absorption of <0.5%, and the low dose of bismuth compounds used to eradicate *H. pylori* typically does not cause neurotoxicity (Alkim et al., 2017).

**FIGURE 1:**
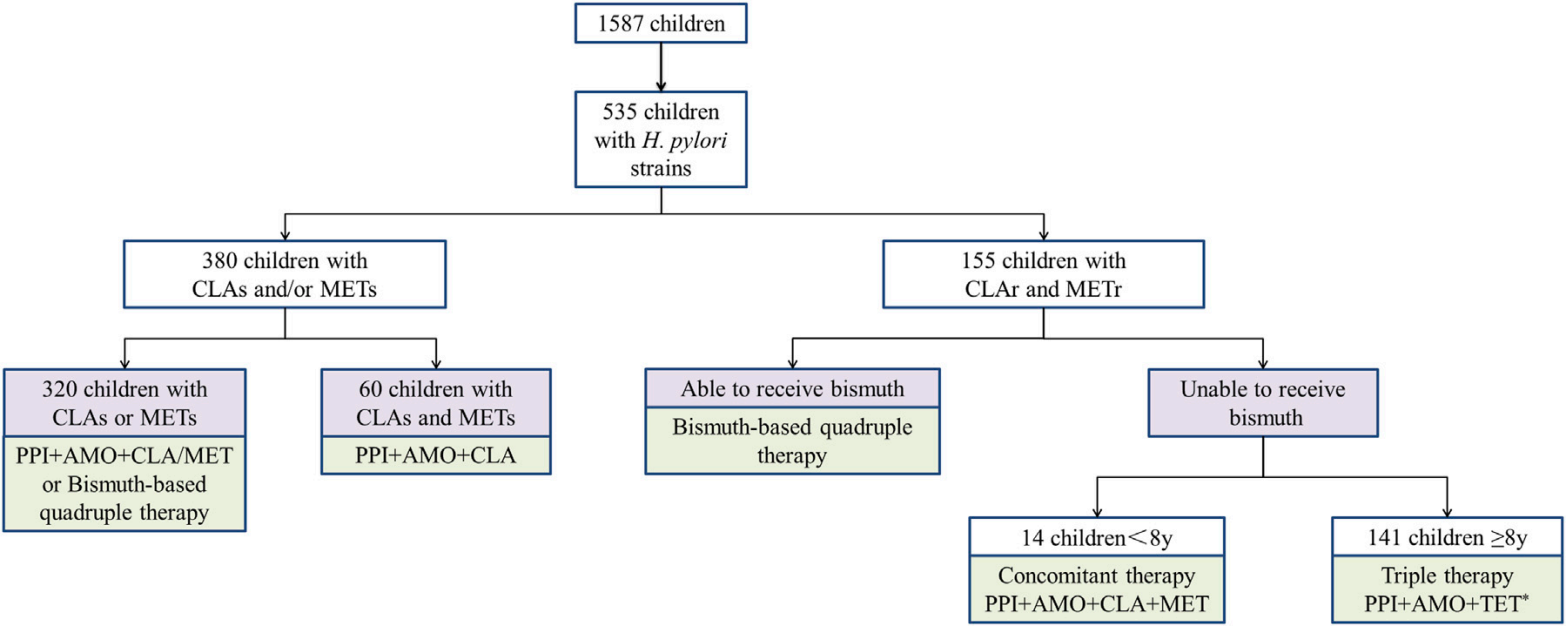
Individualized treatment strategies for *Helicobacter pylori* in Chinese children based on susceptibility testing. * 43 children above 14 years old could use TET or FZD. PPI proton pump inhibitors, AMO amoxicillin, CLA clarithromycin, MET metronidazole, TET tetracycline.

Hence, for children infected with dual-resistant *H. pylori*, bismuth-based quadruple therapy is preferred. If bismuth compounds are not accepted, individualized treatment can be selected based on age (see Figure 1).

### 4.3 Avoid these factors associated with treatment failure

We collected epidemiology and current resistance data on *H. pylori* in children in a specific area. Individualized treatment based on culture tests is a universally acknowledged strategy. However, recent research showed that the eradication rate by therapies based only on susceptibility testing does not reach 90% in children (Le et al., 2022). Insufficient acid-suppressive and poor therapy compliance may be the reasons for failure.

*H. pylori* eradication depends on a combination of acid suppressants and antibiotics. Gastric acid suppressants reduce the minimal inhibitory concentration of antibiotics by increasing the gastric pH and increase the antibacterial drug concentration by diminishing the gastric juice volume. PPIs are commonly used for acid suppression in pediatric eradication treatments. The insufficient acid-suppressive capacity of PPIs is related to under-dosing, ingestion of drugs at inappropriate times (not prior to meals), and genetic polymorphisms of hepatic CYP enzyme activity (Litalien et al., 2005). First-generation PPIs, including lansoprazole, pantoprazole, and omeprazole, and second-generation PPIs, such as dexlansoprazole, are mainly metabolized by CYP2C19 and less metabolized by CYP3A4. More than 90% of omeprazole is metabolized by CYP2C19. Other second-generation PPIs including esomeprazole and rabeprazole are less dependent on CYP2C19, and rabeprazole is mainly eliminated through non-enzymatic mechanisms (El Rouby et al., 2018). The CYP2C19 genotype is associated with PPI exposure and affects drug efficacy and causes adverse reactions. Individuals are divided into extensive metabolizers (EM), intermediate metabolizers (IM), and poor metabolizers (PM), and metabolic phenotypes vary among different regions. Research on Chinese children showed that the proportions of EM, IM, and PM are 38.04%–49.1%, 40.2%–53%, and 10%–10.7%, respectively (Zhang et al., 2020; Zhou et al., 2020; Luo et al., 2023). Extensive metabolism may cause the failure of omeprazole triple therapy to eradicate *H. pylori,* but does not substantially impact rabeprazole triple therapy (Kuo et al., 2014; Lin et al., 2018). Similar conclusions were reported in another study, where the eradication rates of the EM, IM, and PM groups were 60.7%, 84.2%, and 100%, respectively, when the same antibacterial drug was used in combination with omeprazole, whereas this difference did not occur in combination treatment with rabeprazole (Lin et al., 2017). In Europe, esomeprazole (60%) was the most used PPI for tailored triple therapy in children, followed by omeprazole (32%), pantoprazole (6%), and lansoprazole (2%) (Le et al., 2023). In addition, CYP2C19 activity is higher during puberty, and higher doses may be required to achieve ideal acid inhibition (El Rouby et al., 2018). Hence, children should receive higher PPIs doses than the dose administered to adults based on body weight. The updated pediatric guidelines recommend higher PPIs doses for all regimens, with omeprazole doses ranging from 1 to 2 mg/kg/day (Jones et al., 2017; Manfredi et al., 2023). However, omeprazole doses below 1 mg/kg/day have been used for many years in China, and recently updated guidelines for children recommend 1 mg/kg/day for IM. Therefore, it is necessary to consider the CYP2C19 genotype in Chinese children or amend the PPI types used to increase the acid inhibition ability to treat *H. pylori* infection in Chinese children.

Even if a doctor formulates strict and accurate treatment, the treatment is not effective if it is insufficiently implemented in children. Compliance among patients with chronic diseases is often lower than that among those with acute diseases (Osterberg and Blaschke, 2005). Although *H. pylori* infection is not a chronic disease and the treatment duration does not exceed 2 weeks, poor compliance strongly impacts the eradication rate (Shoiab et al., 2018). High compliance is defined as >90% intake of the prescribed dose. Kotilea et al. demonstrated that eradication was achieved in 89.9% of children with high compliance, but in only 36.8% of children with low adherence (Kotilea et al., 2017). In another study, counseling by pharmacists increased medication adherence from 27.5% to 45%, with the eradication rate of *H. pylori* increasing from 28.5% to 42.5% (Shoiab et al., 2023).

Moreover, there is a lack of child-specific preparations of *H. pylori* treatment drugs in China. Tablets or capsules are often needed to distribute the dosage, which leads to an unpleasant taste. In addition to education on diseases and medication counseling from pharmacists, other measures may be needed. A study of non-adherence in *H. pylori* treatment found that 80% of patients missed doses because of forgetfulness (Shakya et al., 2016). Hence, electronic monitoring of medication records and reminders can be used to reduce the number of missed medications.

Therapy compliance includes sufficient prescription dosage and proper medication use to achieve an optimal therapeutic role. Medication for *H. pylori* eradication in children based on body weight is summarized in Table 4. Ingesting antibiotics after meals and PPIs before meals provides a suitable environment for antibiotics to exert their effects. Another therapeutic drug, bismuth, dissolves in the stomach at concentrations of pH four to seven, which can reduce its efficacy. Therefore, bismuth should be used before administering PPIs.

**TABLE 4:** Dosage and usage of oral therapeutic drugs available for *Helicobacter pylori* eradication treatment in children (recommended in China).

Medicine	Dosage	Maximum dosage	Administration
Proton pump inhibitors			
Lansoprazole	0.7–3.3 mg/kg/d, bid	30 mg once	15–30 min before meals
Omeprazole	1 mg/kg/d, bid EM or refractory *H. pylori* infection, 2 mg/kg/d, tid-qid	20 mg once	15–30 min before meals
		20 mg once	
Antibiotics			
Amoxicillin	50 mg/kg/d, bid	1,000 mg once	30 min after meals
Clarithromycin	15–20 mg/kg/d, bid	500 mg once	30 min after meals
Metronidazole	20 mg/kg/d, bid	500 mg once	30 min after meals
Tetracycline	≥8 years old, 25–50 mg/kg/d, tid-qid	500 mg once	30 min after meals
Furazolidone	≥14 years old, 5–10 mg/kg/d, bid	100 mg once	30 min after meals
Bismuth	≥6 years old, 6–8 mg/kg/d, bid	165 mg once	30 min before PPIs

### 4.4 Other developing personalized strategies of *Helicobacter pylori*

Eradication of *H. pylori* infection in children using a combination of antimicrobials and PPIs rarely exceeds 90%; therefore, researchers have investigated alternative strategies, including probiotics, vaccines, and phytomedicines, for *H. pylori* eradication.

#### 4.4.1 Probiotics

The use of antibiotics and PPIs for *H. pylori* eradication alters the gastrointestinal mucosal immune response and intestinal inflammatory microenvironment, resulting in a microbiota imbalance (Manfredi et al., 2023 ). Moreover, initiating *H. pylori* eradication can alter gut microbiota diversity, which may have a long-lasting effect (Sitkin et al., 2022). Hence, supplementation with probiotics and microbial metabolites should be considered to reduce the negative effects of eradication. However, whether probiotics improve the eradication rate of *H. pylori* is unclear. Published studies are inconsistent with respect to the types, dose, timing, and duration of probiotic use. Furthermore, the studies used different eradication therapies. Therefore, the use of probiotics as and adjuvant treatment for *H. pylori* eradication varies by country (Jones et al., 2017; Kato et al., 2019; Kato et al., 2020; Leung et al., 2023). The Maastricht VI/Florence guidelines state that *Lactobacillus*, *Bifidobacterium*, and *S. boulardii* can improve eradication rates in adults (Malfertheiner et al., 2022). However, there is a lack of agreement regarding the effect on eradication of adding single or combination probiotics, especially in children (Daelemans et al., 2022).

#### 4.4.2 Vaccines

The annual recurrence rate of *H. pylori* worldwide is 4.3% (Hu et al., 2017), and is considerably higher in developing countries (13%) than in developed countries (2.7%) (Sun and Zhang, 2019). Although the infection rate of *H. pylori* is decreasing, the recurrence rate has not decreased (Hu et al., 2017). Recurrence is generally associated with severe resistance, which increases the difficulty to treatment. Vaccines have the potential to achieve eradication and prevention (Ding et al., 2018; Wang et al., 2021). Most vaccines are in the early stage of development (Phase I or preclinical studies), and studies have had variable results (Stubljar et al., 2018; Dos Santos et al., 2021). However, the results of a phase III clinical trial of a *H. pylori* subunit vaccine, conducted in children in China aged 6–15 years, showed a 1-year efficacy rate of 71.8% and a 3-year efficacy rate of 65% (Zeng et al., 2015). Vaccines are a promising strategy for use in children because children are infected at a younger age and have a high recurrence rate. However, effective, safe, and immunogenic vaccines are not currently available.

#### 4.4.3 Phytomedicines

Many plant products or their bioactive compounds have antibacterial properties. Several *in vitro* studies have found that certain plant extracts, such as blueberry and grape seed, cinnamon extract, and flavonoids, have anti-*H. pylori* activity (Liu et al., 2018; Sousa et al., 2022). Currently, all *in vivo* studies have been conducted in animals, and no human safety or efficacy studies, or clinical studies on children, are available.

## 5 Conclusion

We observed antibiotic resistance to CLA, MET, AMO, TET, FZD, and LEV in clinical strains isolated from Chinese children between 2019 and 2022. Individualized treatment was designed based on the results of antimicrobial susceptibility testing. The antibiotic resistance rate of *H. pylori* in Chinese children is high; thus, to achieve a 90% eradication rate, it is also necessary to avoid inappropriate use of PPIs and poor therapy compliance. Measures are needed to strengthen the management of antibiotics, reduce unnecessary exposure to antibiotics, and curb the deterioration of drug resistance. Additionally, eradication plans must be standardized to improve the eradication rate.

Based on drug resistance issues and previous studies, we summarized personalized treatment strategies for regional resistance to achieve an initial eradication rate of over 90%. However, there is no practical support for individualized treatment strategy analyses. Therefore, we plan to conduct prospective single or multicenter studies based on these treatment strategies to explore real-world data.

## Data Availability

The original contributions presented in the study are included in the article/supplementary material, further inquiries can be directed to the corresponding authors.
